# Regulation of submaxillary gland androgen-regulated protein 3A via estrogen receptor 2 in radioresistant head and neck squamous cell carcinoma cells

**DOI:** 10.1186/s13046-017-0496-2

**Published:** 2017-02-06

**Authors:** Jennifer Grünow, Chao Rong, Jan Hischmann, Karim Zaoui, Christa Flechtenmacher, Klaus-Josef Weber, Peter Plinkert, Jochen Hess

**Affiliations:** 1 Department of Otolaryngology, Head and Neck Surgery, University Hospital Heidelberg, Im Neuenheimer Feld 400, 69120 Heidelberg, Germany; 2 Institute of Pathology, University of Heidelberg, Heidelberg, Germany; 3 Department of Radiation Oncology, University of Heidelberg, Heidelberg, Germany; 4 Research Group Molecular Mechanisms of Head and Neck Tumors, German Cancer Research Center (DKFZ), Heidelberg, Germany

**Keywords:** ESR2, Estrogen, Fulvestrant, HNSCC, OPSCC, Radiotherapy, SMR3A, Tamoxifen

## Abstract

**Background:**

Molecular mechanisms of intrinsic or acquired radioresistance serve as critical barrier for curative therapy of head and neck squamous cell carcinoma (HNSCC) and remain a major obstacle for progression-free and disease-specific survival.

**Methods:**

HNSCC cell lines were treated with a protocol of fractionated irradiation (IR, 4× 2Gy) alone or in combination with antagonists of estrogen receptor signaling and viability was determined by a colony-forming assay (CFA). Expression of submaxillary gland androgen-regulated protein 3A (SMR3A) and estrogen receptor 2 (ESR2) were assessed in tumor cells in vitro by RQ-PCR, Western blot analysis and immunofluorescence staining, and by immunohistochemical staining of tissue microarrays containing tumor sections from patients with oropharyngeal squamous cell carcinoma (OPSCC), which were treated by definitive or adjuvant radiotherapy. Subgroups with distinct SMR3A and ESR2 expression patterns were correlated with clinical parameters and survival outcome including multivariable analysis.

**Results:**

Fractionated irradiation (IR) revealed an accumulation of tumor cells with prominent SMR3A expression, which was accompanied by an up-regulation of the estrogen receptor 2 (ESR2). ESR2-dependent regulation of SMR3A was supported by induced expression after stimulation with estradiol (E2), which was impaired by co-treatment with 4-Hydroxytamoxifen (TAM) or Fulvestrant, respectively. Both drugs significantly sensitized FaDu cells to fractionated IR as determined by a CFA and accelerated apoptosis. These data suggest a critical role of ESR2 in radioresistance and that SMR3A might serve as a surrogate marker for active ESR2 signaling. In line with this assumption, ESR2-positive oropharyngeal squamous cell carcinoma (OPSCC) with high SMR3A expression had an unfavorable progression-free and disease-specific survival as compared to those tumors with low SMR3A expression.

**Conclusions:**

In summary, our findings provide compelling experimental evidence that HNSCC with SMR3A and ESR2 co-expression have a higher risk for treatment failure and these patients might benefit from clinically well-established drugs targeting estrogen receptor signaling.

**Electronic supplementary material:**

The online version of this article (doi:10.1186/s13046-017-0496-2) contains supplementary material, which is available to authorized users.

## Background

Head and neck cancer is one of the most prevalent human malignancies with an annual incidence of approximately 600,000 new cases worldwide [1]. The majority are head and neck squamous cell carcinoma (HNSCC) originating from the mucosal epithelia of the upper aerodigestive tract. HNSCC is a rather heterogeneous disease and despite aggressive, multimodal treatment of locally advanced tumors a significant proportion of patients develop disease recurrence due to either local or distant failure [2]. Given that many patients with a recurrent HNSCC are no longer amenable to curative therapy, the ensuing morbidity is high and survival is dismal [3]. Consequently, appropriate treatment of HNSCC patients remains a major challenge and there is an urgent demand in a better understanding of molecular principles underlying treatment failure.

In the past, we applied global gene expression profiling on matched samples of primary and recurrent tumors of an orthotopic squamous cell carcinoma model in mice to identify differentially expressed genes [4]. One candidate gene with an increased transcript level in recurrent as compared to primary tumors encoded for the mouse homologue of human submaxillary gland androgen-regulated protein 3A (SMR3A), which belongs to the opiorphin gene family [5]. More recently, SMR3A expression was detected in a subgroup of patients with primary oropharyngeal squamous cell carcinoma (OPSCC) and served as an independent risk factor for unfavorable prognosis [6]. However, the regulation of SMR3A and its putative mode of action in the pathogenesis of HNSCC or in response to treatment have not been addressed, so far.

In the current study, we demonstrate prominent SMR3A expression in tumor cells upon fractionated irradiation (IR) and provide experimental evidence that induced SMR3A expression serves as a surrogate maker for active estrogen receptor 2 (ESR2) signaling in radioresistant tumor cells.

## Methods

### Cell culture and stable clones

Human cell lines (FaDu and Cal27) were purchased from ATCC (http://www.lgcstandards-atcc.org/) and were maintained in Dulbecco’s Modified Eagle’s Medium (Sigma-Aldrich, Germany) supplemented with 10% fetal bovine serum (Invitrogen, Germany), 2 mM L-Glutamine (Invitrogen, Germany) and 50 μg/ml Penicillin-Streptomycin (Invitrogen, Germany) in a humidified atmosphere of 6% CO_2_ at 37 °C. Authentication of both cell lines was confirmed by the Multiplex Human Cell Line Authentication Test (Multiplexion, Germany, latest update August 11th, 2016). To generate single cell clones, FaDu cells were transfected with either pcDNA3.1 (Invitrogen, USA) or a plasmid encoding a Myc-DDK-tagged ORF of Homo sapiens SMR3A (Origene, USA) using FuGene HD Transfection Reagent (Promega, Germany) according to the manufacturer’s instruction. Following selection with 0.5–0.8 mg/ml G418 (Calbiochem Merck, Germany) for two weeks, single clones were isolated and ectopic SMR3A-Myc/DDK expression was confirmed on transcript and protein level. Two independent mock controls and FaDu-SMR3A clones were selected for further analysis. Parental FaDu and Cal27 cells were treated with the indicated concentrations of estradiol (E2) or 4-Hydroxytamoxifen (TAM) dissolved in ethanol (EtOH) or Fulvestrant dissolved in DMSO. All compounds were purchased from Sigma-Aldrich, Germany.

### RQ-PCR analysis

RNA extraction, cDNA synthesis and real-time quantitative polymerase chain reaction (RQ-PCR) were done as described previously [7]. Primer sequences were selected using Primer-Blast (http://www.ncbi.nlm.nih.gov/tools/primer-blast) and are listed in Additional file 1. To quantify relative SMR3A transcript levels by RQ-PCR analysis cDNA samples were used undiluted. The cycle of threshold (CT) of the gene of interest was standardized to the CT value of the reference gene (LMNB1) using the ∆∆CT method.

### Western blot analysis and immunofluorescence staining

Western blot analysis was performed with whole cell lysates as described previously [8]. Membranes were incubated in enhanced chemiluminiscence solution (Thermo Scientific, Germany) and developed with the ImageQuant LAS500 system (GE Healthcare, Germany). Immunofluorescence staining was done as described elsewhere [7], and pictures were taken with the Fluorescence Microscope BX50F, Olympus XC30 Camera and cellSens Entry imaging software (Olympus, Germany). Antibodies and dilutions that were used for Western blot analysis and immunofluorescence staining are listed in Additional file 2.

### BrdU-incorporation assay

20–30,000 cells per well were seeded on glass coverslips in 12-well plates. 48 h after seeding, cells were treated for 2 h with BrdU according to the manufacture’s instruction (BD Pharmingen, Germany). Staining was done as described previously [7, 8] and relative amount of proliferative cells was calculated as the ratio of BrdU-positive versus total cell counts.

### Migration assay

Cell migration assays were performed using ibidi Culture Inserts (Ibidi, Germany) according to the instruction of the manufacturer. After removal of the inserts, pictures were taken at the indicated time points using the Keyence BZ-9000 microscope system to calculate the relative gap closure over time.

### Colony-formation assay (CFA)

To determine the clonal expansion of tumor cells upon fractionated irradiation, 100, 300 and 1,000 cells were seeded per well in 6-well plates and irradiated on four consecutive days with a daily dose of 2 gray (Gy) using X-RAD 320 (Precision X-Ray, North Branford, CT USA) or kept untreated as controls. Half of the cells were treated daily with 1 μM 4-Hydroxytamoxifen (TAM, Sigma-Adrich, Germany) or every second day with 30 nM Fulvestrant (Sigma-Adrich, Germany). After 10–14 days in culture, clones were stained with crystal violet and total amount of colonies was quantified as described in [9]. The survival fraction was computed according to [10].

### Patient samples and immunohistochemistry

The retrospective study cohort, generation of tissue microarrays, immunohistochemical staining and assessment of the immunoreactivity score for SMR3A was described before [6]. Paraffin-embedded tissue specimens were provided by the tissue bank of the National Center for Tumor Disease (Institute of Pathology, University Hospital Heidelberg) after approval by the local institutional review board (ethic votes: 176/2002 and 206/2005). The study was performed according to the ethical standards of the Declaration of Helsinki. For all tumor samples, clinical and follow-up data were available from the Department of Otolaryngology, Head and Neck Surgery at the University Hospital Heidelberg. Immunohistochemical staining for ESR2 was done using the 3, 3’-diaminobenzidine (DAB) peroxidase substrate kit (Perkin Elmer, Germany) according to the manufacturer’s instruction and the assessment of the immunoreactivity score for ESR2 was according to the procedure described before [6]. Clinical and histopathological features of samples for which informative values for SMR3A and ESR2 were available and which were included in the final analysis are listed in Additional file 3.

### Statistical analysis

Statistical analysis was done using SPSS (version 21) and GraphPad software (http://www.graphpad.com). Differences between patient subgroups were assessed using Chi square test. Disease-specific survival (DSS) was calculated as the time from the date of primary tumor diagnosis to the date of cancer-related death within the follow-up interval (events). Survival times of patients, who were alive or were dead due to causes other than cancer were censored. Progression-free survival (PFS) was calculated from the date of primary tumor diagnosis to the date of the first local recurrence, lymph node or distant metastasis, second primary carcinoma, or date of cancer-related death within the follow-up period (events). Patients without progression (no event) or cancer-unrelated death were censored. The method of Kaplan–Meier was used to estimate survival distributions and differences between subgroups were determined by log-rank tests. To adjust for possible confounders, multivariable Cox proportional hazard regression models were fitted. Models included the covariates gender, age, clinical staging, alcohol and tobacco consumption, HPV status and therapy. Based on availability of complete clinical data sets, 66 cases were included for the analysis of subgroups with ESR^high^SMR3A^low^ versus ESR^high^SMR3A^high^ staining patterns and 103 cases were included for the analysis of subgroups with ESR^high^SMR3A^low^ versus all other staining patterns.

In all statistical tests, a *p*-value of 0.05 or below was considered as statistically significant.

## Results

### Establishment and analysis of a HNSCC cell line with ectopic SMR3A expression

SMR3A expression was assessed by RQ-PCR analysis with cDNA from human HNSCC cell lines. Transcript levels were close to the detection limit in all cell lines tested, indicating no or a rather low SMR3A expression under normal growth conditions (data not shown). FaDu cells were selected to generate stable clones (FaDu-SMR3A) with ectopic SMR3A expression in order to address its impact on tumor-relevant processes in vitro. Ectopic expression in FaDu-SMR3A clones was confirmed on transcript and protein levels (Additional file 4a-c). However, we did not observe any significant difference in tumor cell proliferation or migration between FaDu-SMR3A clones and mock controls (Additional file 4d-e). In summary, these data suggested that SMR3A has no major impact on tumor cell physiology under normal growth conditions but raised the attractive question, whether it serves as a marker for a distinct subpopulation of tumor cells with higher resistance against well-established treatment options.

### SMR3A expression in HNSCC cells upon fractionated irradiation

**Fig. 1:**
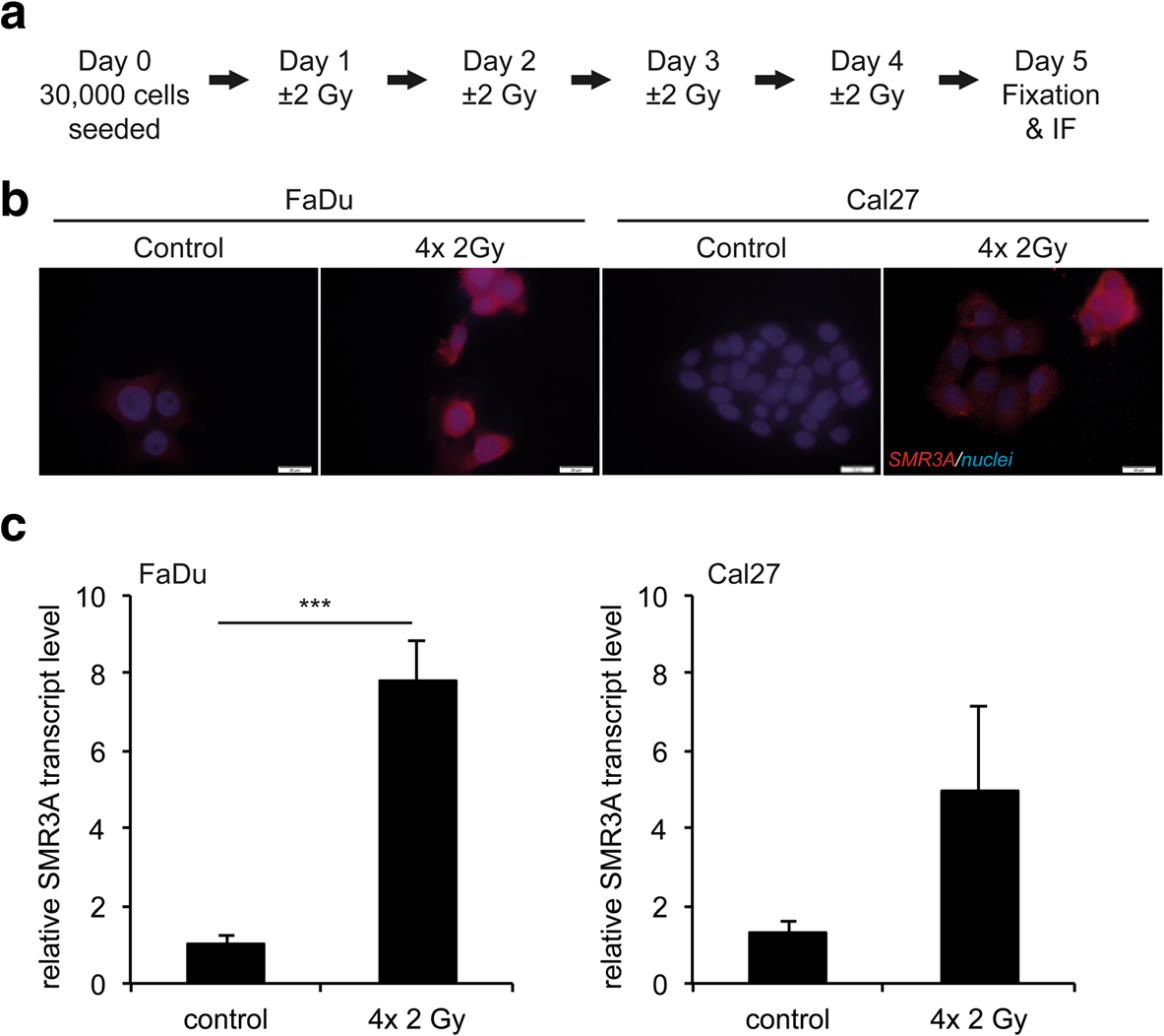
SMR3A expression in HNSCC cell lines after fractionated IR. **a** Schematic summary of the treatment protocol for fractionated IR. Prominent SMR3A expression in FaDu and Cal27 cells after fractionated IR (4× 2Gy) was demonstrated by immunofluorescence staining on protein level **b **(*red signal*) and by RQ-PCR on transcript level (**c**) Cell nuclei were counterstained with Hoechst H33342 (*blue signal*). Scale bars = 20 μm. Bars represent mean values ± SEM of two independent experiments measured in triplicates with quantification of LMNB1 transcript levels as reference gene. *** *p* ≤0.0005

To support this assumption, FaDu cells were treated with fractionated irradiation (IR, 4× 2Gy), which revealed a prominent SMR3A staining in all vital cells after treatment as determined by IF analysis (Fig. 1a-b). It is worth noting that we observed a gradual increase in the relative amount of positive cells with increasing cycles of fractionated IR (Additional file 5a and data not shown). An induction after fractionated IR was also detected in Cal27 cells, though the staining was more heterogeneous and not all vital cells were SMR3A-positive (Fig. 1b). In both cell lines induced SMR3A expression after fractionated IR was confirmed on transcript level (Fig. 1c). However, the induction as compared to control-treated cells was highly significant for FaDu cells, while in Cal27 cells a clear trend was found without reaching statistical significance.

### SMR3A is a downstream target of ESR2 signaling

**Fig. 2:**
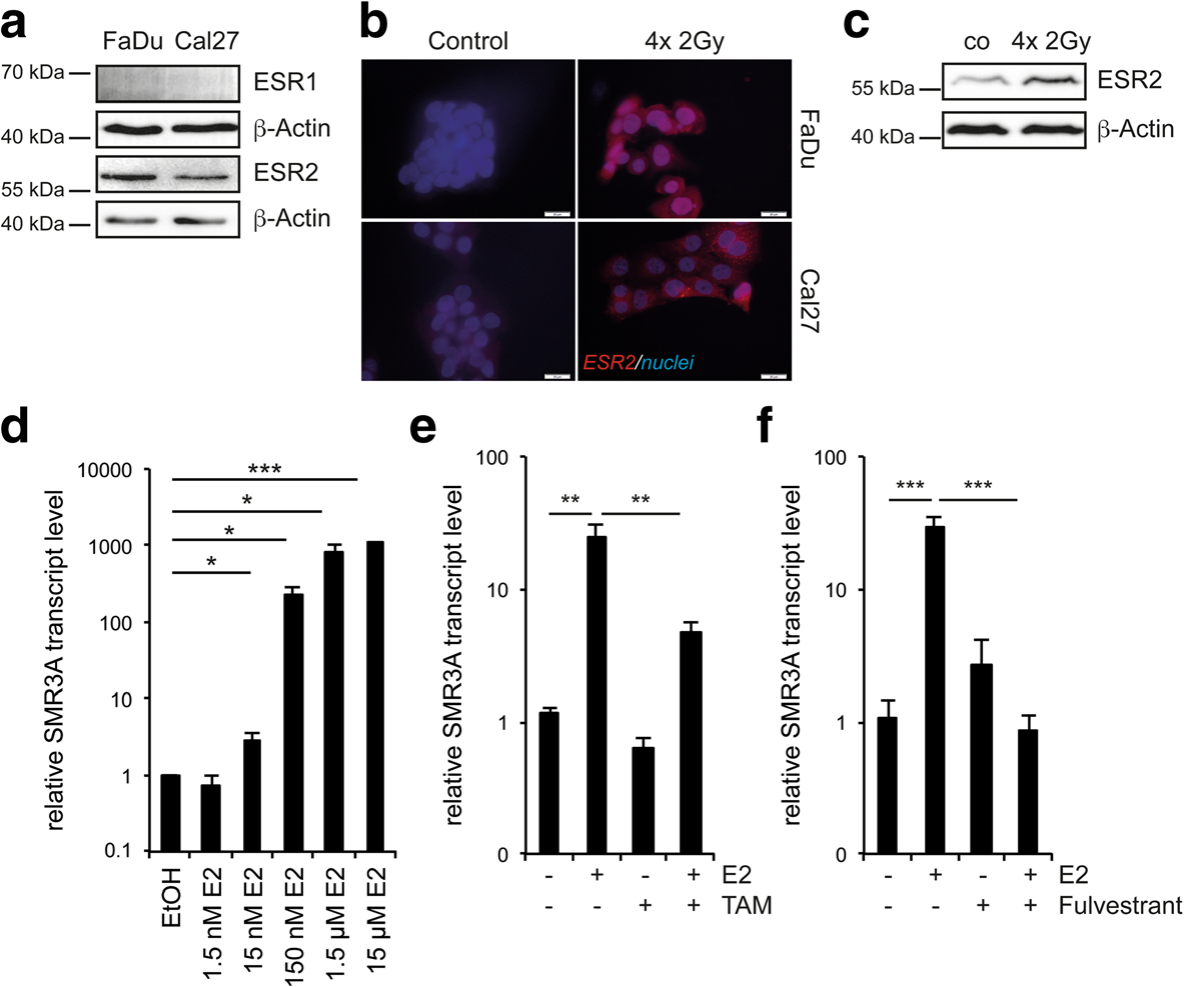
Regulation of SMR3A expression by ESR2 signaling. Western blot analysis revealed basal expression of ESR2 but not ESR1 protein in tumor cell lines (**a**) which was induced by fractionated IR as demonstrated by immunofluorescence staining **b** (*red signal*). Cell nuclei were counterstained with Hoechst H33342 (*blue signal*). Scale bars = 20 μm. Induced ESR2 protein expression after fractionated IR was confirmed in FaDu cells by Western blot analysis (**c**). Detection of β-Actin served as a control for protein quality and quantity. RQ-PCR revealed concentration dependent induction of relative SMR3A transcript levels by E2 in FaDu cells (**d**) which was impaired by administration of 1 μM TAM (**e**) or 10 nM Fulvestrant (**f**) respectively. Bars represent mean values ± SEM of at least two independent experiments measured in triplicates with quantification of LMNB1 transcript levels as reference gene. * *p* ≤ 0.05, *** *p* ≤ 0.0005

In the past, several studies in rodents provided experimental evidence for the regulation of opiorphin family members by steroid hormones [11–13]. To assess whether hormone signaling also contributes to SMR3A regulation during fractionated IR, expression of the androgen receptor (AR) as well as estrogen receptor 1 and 2 (ESR1 and ESR2) was analyzed in FaDu and Cal27 cells on transcript and protein levels. No AR and ESR1 expression was detected under normal growth conditions and only few cells exhibited a minor staining for ESR1 after fractionated IR (Fig. 2a, Additional file 5b and data not shown). In contrast, ESR2 was expressed in both cell lines and was strongly induced after fractionated IR (Fig. 2a-c). Again, the amount of ESR2-positive cells was more heterogeneous in Cal27 as compared to FaDu cells and accumulated with increasing cycles of fractionated IR (Additional file 5a), suggesting regulation of SMR3A expression by ESR2-dependent signaling. In line with this assumption, stimulation of FaDu cells with estradiol (E2) revealed a significant and concentration dependent increase of SMR3A transcript levels (Fig. 2d), which was impaired by co-treatment with 4-Hydroxytamoxifen (TAM) or pre-treatment with Fulvestrant (Fig. 2e-f). In summary, these data suggested that SMR3A expression serves as a potential surrogate marker for active ESR2 signaling in a subpopulation of radioresistant tumor cells, raising the question, whether pharmacological interference sensitizes these cells to fractionated IR.

### Inhibition of ESR2 signaling in combination with fractionated IR

**Fig. 3:**
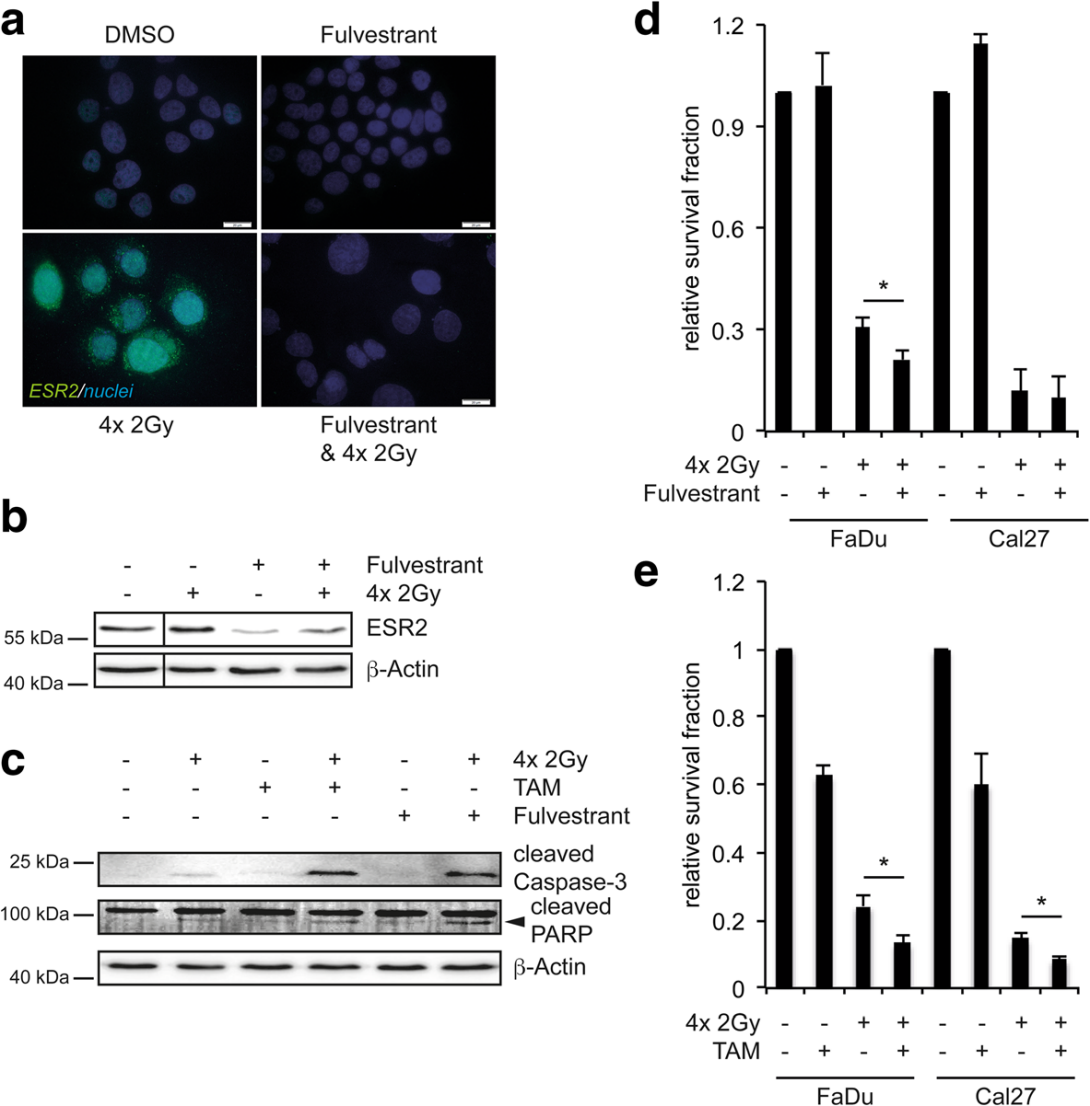
Impact of TAM or Fulvestrant treatment on fractionated IR of FaDu cells. **a** Representative pictures of an immunofluorescence staining of control (DMSO) and 10 nM Fulvestrant-treated FaDu cells with or without fractionated IR (4× 2Gy) demonstrate reduced basal and impaired induction of ESR2 protein levels (*green signal*) by Fulvestrant, which is confirmed by Western blot analysis (**b**) Cell nuclei were counterstained with Hoechst H33342 (*blue signal*). **c** Western blot analysis indicates accelerated apoptosis by the combination of fractionated IR with 1 μM TAM or 10 nM Fulvestrant, respectively, as determined by increased levels of cleaved caspase three and PARP (indicated by the *arrowhead*). Detection of β-Actin served as a control for protein quality and quantity. **d**-**e** Graphs represent the relative survival fraction of FaDu and Cal27 cells in a colony-forming assay after fractionated IR (4× 2Gy) and either 30 nM Fulvestrant or 1 μM TAM administration, respectively. Control-treated cells are set to one and *bars* represent mean values ± SEM of at least three independent experiments. * *p* ≤ 0.05

As a proof of concept, fractionated IR was applied to FaDu cells with or without administration of TAM or Fulvestrant, respectively (Additional file 6). Fulvestrant inhibited irradiation-induced ESR2 expression as determined by IF staining and Western blot analysis (Fig. 3a-b), and augmented apoptosis, which was monitored by elevated caspase three and PARP cleavage (Fig. 3c). An increase in apoptosis was also detected for administration of TAM in combination with fractionated IR (Fig. 3c). A higher radiosensitivity of FaDu cells by inhibition of ESR2 signaling was further confirmed in a colony-forming assay after fractionated IR alone or in combination with Fulvestrant or TAM treatment (Fig. 3d-e). In line with the less efficient induction of ESR2 and SMR3A, Cal27 exhibited a reduced relative survival fraction after fractionated irradiation as compared to FaDu cells, suggesting a positive correlation between ESR2 induction and radioresistance. Furthermore, in Cal27 cells only administration of TAM but not Fulvestrant revealed a significant decrease in the relative survival fraction upon fractionated IR.

### Co-expression of ESR2 and SMR3A in tumor samples and correlation with clinical features

**Fig. 4:**
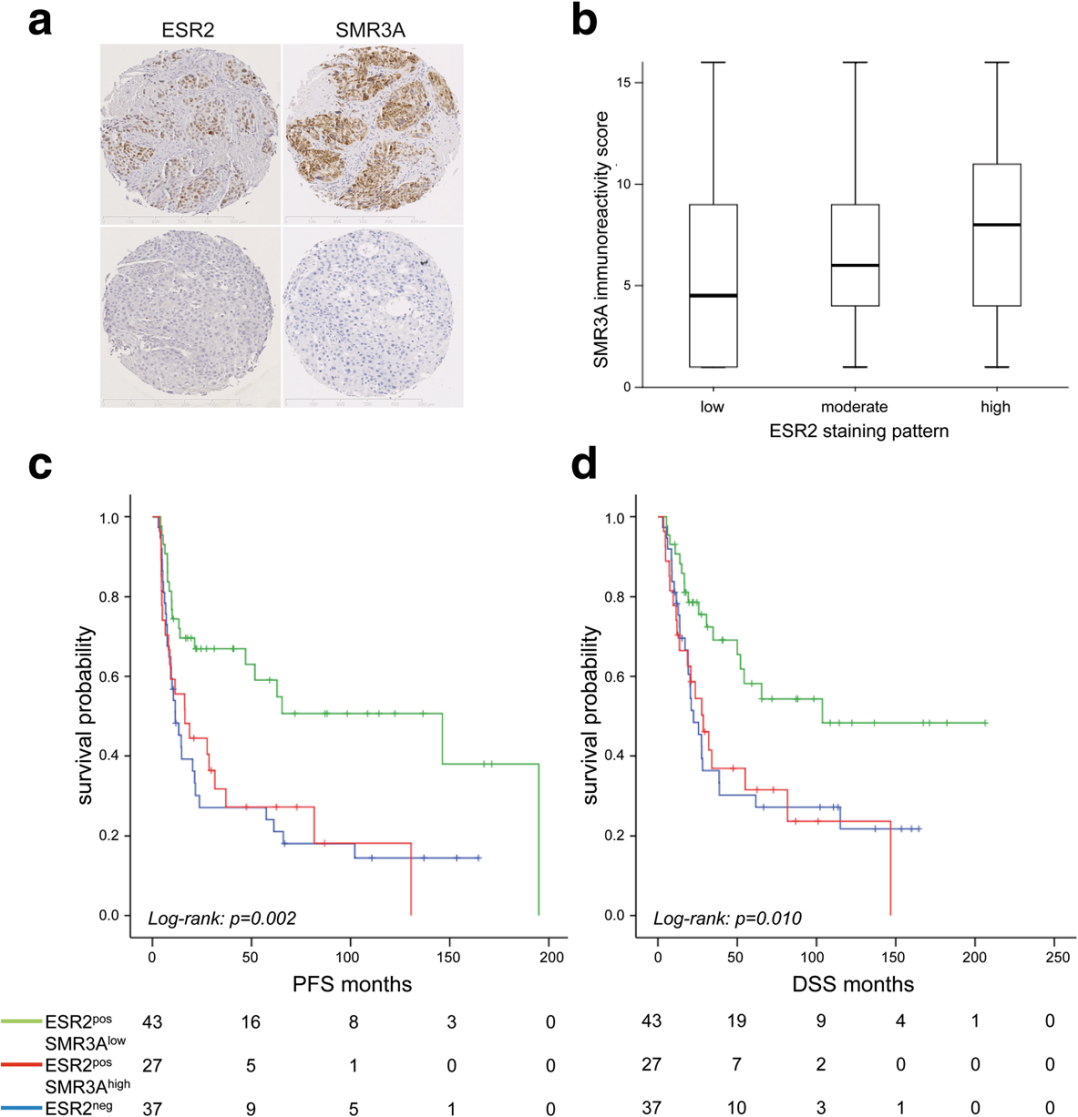
SMR3A and ESR2 expression in HNSCC patients. **a** Representative pictures of immunohistochemical staining (*brown signal*) of serial tumor sections with anti-ESR2 (*left row*) or anti-SMR3A antibodies (*right row*). Haematoxyline counterstaining (*blue staining*) demonstrates the tissue architecture. Scale bars = 500 μM. **b** Boxblot depicts the SMR3A immunoreactivity score as mean value and 5th/95th percentile for individual tumors with low, moderate or high ESR2 staining pattern. **c-d** Kaplan-Meier graphs show differences in disease-specific (DSS) and progression-free survival (PFS) between subgroups without detectable ESR2 staining (ESR2^neg^, *blue line*) and ESR2-positive tumors with low (*green line*) or high SMR3A expression (*red line*)

So far, experimental data support a model in which ESR2 and SMR3A co-expression after fractionated IR is a characteristic feature for a subpopulation of treatment resistant tumor cells. In line with this model the detection of SMR3A expression in tumor cells prior to therapy might serve as a surrogate marker for active ESR2 signaling during malignant progression and the presence of tumor cells with intrinsic radioresistance. To address the clinical relevance of our in vitro findings, we assessed ESR2 expression by IHC staining on tissue microarrays containing tumor specimens of OPSCC patients, which were treated with either definitive or post-surgical radiotherapy with or without adjuvant chemotherapy. Data on SMR3A staining on serial sections were already available from a previous retrospective study [6]. Evaluable staining patterns for both proteins were obtained for *n* = 109 OPSCC patients (Fig. 4a), and clinical features of the study cohort are summarized in Additional file 3. Positive staining for ESR2 (ESR2^pos^) in tumor cells was detected in 65.1% of tumors and a high staining pattern correlated significantly with a higher SMR3A immunoreactivity score as compared to samples without detectable ESR2 staining (Fig. 4b, *p* = 0.044). We did not observe a statistically significant correlation between ESR2^neg^ and ESR2^pos^ subgroups and patient characteristics tested, except for age, T status and tobacco consumption. Significant associations were due to an older age, smaller tumor size and an enrichment of never/former smokers in the subgroup with ESR2^pos^ staining (Additional file 7). Concerning progression-free (PFS) and disease-specific survival (DSS), univariate analysis revealed an unfavorable clinical outcome in the absence of ESR2 staining as compared to ESR2^pos^ tumors (Fig. 4c-d). However, patients with ESR2^pos^ tumors had a significantly shorter PFS and DSS in the presence of high SMR3A expression, similar to the ESR2^neg^ subgroup, while ESR2^pos^SMR3A^low^ tumors exhibited the most favorable clinical outcome (Fig. 4c-d, Additional file 8). Multivariable Cox proportional hazard regression models adjusted for the covariates gender, age, clinical staging, alcohol and tobacco consumption, HPV status and therapy supported a favorable PFS and DSS of the subgroup with an ESR2^high^SMR3A^low^ staining as compared to either ESR2^high^SMR3A^high^ or all other staining patterns (Additional file 9).

## Discussion

Radiotherapy remains a mainstay of local treatment for HNSCC and improvements in intensity-modulated techniques and new protocols of altered fractionation have contributed to reduced mortality and long-term morbidity with a strong impact on the quality of life [14]. However, intrinsic and acquired resistance remains a major obstacle and serves as a critical barrier for curative treatment of cancer patients, including HNSCC [15]. Unraveling molecular principles of radioresistance and subsequent clonal expansion of vital tumor cells is a crucial task to establish prognostic biomarkers for HNSCC patients with a higher risk for treatment failure and to identify new drug targets for more efficient and less toxic therapy.

In this study, we unraveled prominent SMR3A expression in vital tumor cells upon fractionated IR. Although ectopic SMR3A expression had no significant impact on tumor-relevant processes under normal growth conditions in vitro, presented data provide compelling experimental evidence that it might serve as a surrogate marker for a subpopulation of resistant cells as a putative source for tumor relapse after radiotherapy. In line with this assumption, a previous retrospective study unraveled high SMR3A expression as a risk factor for unfavorable progression-free and overall survival in a cohort of OPSCC patients [6].

Studies in rodents revealed a positive regulation of opiorphin family members by hormone signaling [11–13], and our findings indicate that ESR2 signaling not only induces SMR3A expression but also plays a critical role in resistance to treatment. This assumption is further supported by recent reports demonstrating that the presence of ESR2 modulates response to several therapeutic agents in breast and lung cancer cells [16–18]. So far, only a limited number of studies focused on estrogen receptor signaling in the pathogenesis or prognosis of HNSCC. Already in 1988, Somers and colleagues demonstrated that estrogen treatment potentiated growth of laryngeal tumors in a xenograft model in vivo [19]. More recently, a functional crosstalk between estrogen and EGF receptor signaling was reported, which might contribute to neoplastic transformation and disease progression of HNSCC [20]. Conflicting data were published with regard to the expression pattern of ESR1 and ESR2 in HNSCC and their correlation with clinical outcome [20–23]. However, in line with our data two studies found a frequent expression of ESR2 but not ESR1 in HNSCC cell lines and tumor tissues [21, 22], suggesting a more prominent role of ESR2-related signaling.

In contrast to ESR1, ESR2 is usually described as a tumor suppressor in estrogen-sensitive malignancies [24]. However, we detected a positive staining for ESR2 expression as a common event in OPSCC, while ESR1-positive tumor cells were a rather rare event (data not shown). In line with our hypothesis that SMR3A serves as a surrogate marker for active ESR2 signaling and predicts treatment resistance, the combined expression of both proteins was associated with an unfavorable clinical outcome concerning progression-free and disease-specific survival after definitive or adjuvant radiotherapy. Although multivariable Cox proportional hazard regression models supported the assumption that ESR2 and SMR3A staining patterns are associated with clinical outcome, our retrospective study is limited by the low amount of patients for distinct subgroups. A major challenge for the future will be the analysis of ESR2 and SMR3A but also other downstream targets in larger cohort studies, including specimens from HNSCC of other locations.

It is worth noting that OPSCC without detectable ESR2 expression were also correlated with a poor survival, indicating that the prognostic value of ESR2 expression is context dependent and that ESR2-negative and positive tumors represent two distinct subgroups of HNSCC which most likely differ in their cellular and molecular traits. In the past, the prognostic value of ESR2 expression in other human malignancies, including breast, ovarian, bladder, prostate and lung cancer, was controversially discussed further supporting a strong context dependency [17, 25–33]. Nevertheless, a negative staining in a tumor biopsy taken at the time point of diagnosis or during primary surgery does not exclude induction and/or expansion of ESR2-positive tumor cells during fractionated radiotherapy. Innovative tools, such as irradiation of ex vivo cultures derived from vital tumor tissue [34, 35], might be appropriate pre-clinical models to address this issue and could help to select individual patients for new treatment options.

## Conclusions

In summary, our data suggest that HNSC’C with a combined ESR2 and SMR3A expression are at a higher risk for treatment failure upon radiotherapy, but might benefit from treatment with TAM or Fulvestrant, two clinically well-established inhibitors targeting estrogen receptor signaling [36]. Indeed, both drugs significantly sensitized tumor cells to fractionated IR and an accelerated sensitivity to chemotherapy after administration of Fulvestrant was demonstrated recently for of estrogen receptor positive breast and lung cancer cells [37, 38]. However, additional cohort studies and preclinical models are required to confirm the clinical relevance of our findings and as a proof-of-concept for the efficacy of TAM or Fulvestrant in combination with radiotherapy for HNSCC patients.

## Additional files

Additional file 1: List of primer sequences for RT-PCR analysis. (DOCX 36 kb)
Additional file 2: List of primary (A) and secondary antibodies (B) for Western blot analysis, immunofluorescence and immunohistochemical staining. (DOCX 82 kb)
Additional file 3: Clinical features of OPSCC patients. (DOCX 54 kb)
Additional file 4: No impact of ectopic SMR3A expression in FaDu cells on tumor-relevant processes under normal growth conditions. (a) Graph represents relative SMR3A transcript levels as determined by RQ-PCR with cDNA from parental FaDu cells (set to one), two mock controls (Mock #1 and #2) and two clones with stable SMR3A transgene expression (SMR3A#1 and #2) derived thereof. Bars represent mean values of an experiment performed in triplicates with quantification of LMNB1 transcript levels as reference gene. SMR3A-Myc/DDK transgene expression in FaDu-SMR3A clones was confirmed by Western blot analysis using an anti-Myc antibody (**b**) and immunofluorescence staining (red signal) with an anti-SMR3A antibody (**c**). Detection of β-Actin served as control for protein quantity and quality, and cell nuclei were visualized by counterstained with Hoechst H33342 (blue signal). Scale bars = 20 μm. No significant differences were detectable between FaDu-Mock and FaDu-SMR3A clones considering proliferation as determined by BrdU incorporation (**d**) or migration as determined by an ibidi Culture Inserts assay (**e**). Graphs represent mean values ± SEM of three independent experiments. (TIF 7232 kb)
Additional file 5: Co-expression of SMR3A and ESR2 in FaDu cells after fractionated IR. Representative pictures of immunofluorescence staining for control and irradiated FaDu cells demonstrate prominent SMR3A and ESR2 expression **a** (red signal), but no positive staining for ESR1 or AR **b** Cell nuclei were visualized by counterstained with Hoechst H33342 (blue signal). Scale bars = 20 μm. (TIF 16501 kb)
Additional file 6: Schematic summary of the treatment protocol for fractionated IR in combination with 4-Hydroxytamoxifen (TAM, a) or Fulvestrant (Fulv, b). (TIF 277 kb)
Additional file 7: Correlation of ESR2 expression with histopathological and clinical characteristics. (DOCX 70 kb)
Additional file 8: Univariate Cox regression models for progression-free and disease-specific survival. (DOCX 77 kb)
Additional file 9: Multivariable Cox regression models for progression-free and disease-specific survival. (DOCX 107 kb)

## Abbreviations

AR: Androgen receptor
DSS: Disease-specific survival
E2: 17-β-estradiol
ESR1: Estrogen receptor 1
ESR2: Estrogen receptor 2
HNSCC: Head and neck squamous cell carcinoma
IR: Irradiation
OPSCC: Oropharyngeal squamous cell carcinoma
PFS: Progression-free survival
SMR3A: Submaxillary gland androgen-regulated protein 3A
TAM: 4-Hydroxytamoxifen
