# Supplementary material for: Regulation of submaxillary gland androgen-regulated protein 3A via estrogen receptor 2 in radioresistant head and neck squamous cell carcinoma cells
Source: J Exp Clin Cancer Res. 2017 Feb 6;36:25. doi: 10.1186/s13046-017-0496-2 (PMC5294868; doi:10.1186/s13046-017-0496-2)
Supplement: Additional file 1: — List of primer sequences for RT-PCR analysis. (DOCX 36 kb) [file 13046_2017_496_MOESM1_ESM.docx]

**Additional file 1.** List of primer sequences for RT-PCR analysis

| **Gene Symbol** |  | **Sequence** | **Annealing temperature [°C]** |
| --- | --- | --- | --- |
| *SMR3A* | for | CTCCTCCACCATGTTTTC | 60 |
|  | rev | GAGTGTGATTGAATTCT |  |
| *LMNB1* | for | GCTGCTCCTCAACTATGCTAAGAA | 60 |
|  | rev | TTTGACGCCCAGAATCCAC |  |
