# Supplementary material for: Regulation of submaxillary gland androgen-regulated protein 3A via estrogen receptor 2 in radioresistant head and neck squamous cell carcinoma cells
Source: J Exp Clin Cancer Res. 2017 Feb 6;36:25. doi: 10.1186/s13046-017-0496-2 (PMC5294868; doi:10.1186/s13046-017-0496-2)
Supplement: Additional file 2: — List of primary (A) and secondary antibodies (B) for Western blot analysis, immunofluorescence and immunohistochemical staining. (DOCX 82 kb) [file 13046_2017_496_MOESM2_ESM.docx]

**Additional file 2.** List of primary (**A**) and secondary antibodies (**B**) for Western blot analysis, immunofluorescence and immunohistochemical staining

| A | Cat. No. | Clone & Species | Company | Application | Dilution |
| --- | --- | --- | --- | --- | --- |
| AR | ab9474 | AR441, monoclonal mouse | Abcam | WB  IF | 1:1,000  1:100 |
| ESR1 | M7047 | 1D5; monoclonal mouse | Dako | WB | 1:1,000 |
| ESR1 | ab3575 | polyclonal rabbit | Abcam | IF | 1:100 |
| ESR2 | ab133467 | EPR3778; monoclonal rabbit | Abcam | WB  IHC  IF | 1:1,000  1:1,000  1:50 |
| SMR3A | ab97942 | polyclonal rabbit | Abcam | IHC  IF | 1:25  1:50 |
| Cleaved-caspase3 (Asp175) | #9661 | Polyclonal rabbit | Cell Signaling | WB | 1:1,000 |
| PARP | #9542 | Polyclonal rabbit | Cell Signaling | WB | 1:1,000 |
| β-Actin | A5441 | AC-15; monoclonal mouse | Sigma-Aldrich | WB | 1:10,000 |
| BrdU  Alexa555 | B35131 | MoBU-1; monoclonal mouse | Thermo Fisher | IF | 1:1,000 |
| DYKDDDDK-Tag | #2368 | 9E10; polyclonal rabbit | Cell Signaling | WB | 1:1,000 |

*WB = Western blot, IHC = immunohistochemistry, IF = immunofluorescence*

| B | Cat. No | Species | Company | Application | Dilution |
| --- | --- | --- | --- | --- | --- |
| anti-mouse-HRP | sc-2005 | Goat | Santa Cruz | WB | 1:10,000 |
| anti-rabbit-HRP | sc-2317 | Donkey | Santa Cruz | WB | 1:10,000 |
| anti-rabbit-Biotin | BA-1000 | Goat | Vector Laboratories | IHC | 1:200 |
| anti-mouse-Alexa488 | 111-545-003 | Goat | Dianova | IF | 1:200 |
| anti-rabbit-Cy3 | 111-165-008 | Goat | Dianova | IF | 1:200 |
| anti-mouse-Cy3 | 715-165-151 | Donkey | Dianova | IF | 1:200 |
| ImmPress® goat |  | Horse | Vector | IHC |  |
| ImmPress® mouse |  | Goat | Vector | IHC |  |

*WB = Western blot, IHC = immunohistochemistry, IF = immunofluorescence*
