# Supplementary material for: Regulation of submaxillary gland androgen-regulated protein 3A via estrogen receptor 2 in radioresistant head and neck squamous cell carcinoma cells
Source: J Exp Clin Cancer Res. 2017 Feb 6;36:25. doi: 10.1186/s13046-017-0496-2 (PMC5294868; doi:10.1186/s13046-017-0496-2)
Supplement: Additional file 3: — Clinical features of OPSCC patients. (DOCX 54 kb) [file 13046_2017_496_MOESM3_ESM.docx]

**Additional file 3. Clinical features of OPSCC patients**

| **Features** | **Category** | **N** | **%** |
| --- | --- | --- | --- |
| Age [years] | 38.4-85.5 (median = 57.6) |  |  |
| Gender | Male | 83 | 76.1 |
|  | Female | 26 | 23.9 |
| T status | T1-T2 | 43 | 39.4 |
|  | T3-T4 | 66 | 60.6 |
| N status | N0 | 12 | 11.0 |
|  | N+ | 97 | 89.0 |
| M status | M0 | 101 | 92.7 |
|  | M+ | 6 | 5.5 |
|  | Mx | 2 | 1.8 |
| Pathological grading | G1-2 | 52 | 56.5 |
|  | G3 | 40 | 43.5 |
|  | missing | 17 |  |
| Clinical staging | I-III | 29 | 26.6 |
|  | IV | 80 | 73.4 |
| Alcohol | no/former | 19 | 17.4 |
|  | current | 90 | 82.6 |
| Tobacco | no/former | 27 | 24.8 |
|  | current | 82 | 75.2 |
| HPV | non-related^1^ | 81 | 77.1 |
|  | related^2^ | 24 | 22.9 |
|  | missing | 4 |  |
| Therapy | adjuvant RT | 54 | 49.5 |
|  | adjuvant RCT | 17 | 15.6 |
|  | definitive RT | 18 | 16.5 |
|  | definitive RCT | 20 | 18.3 |

*RT, radiotherapy, RCT, radiochemotherapy, ^1^ viral DNA-negative or DNA-positive but transcript-negative; ^2^ viral DNA- and transcript-positive according to Holzinger et al., 2012.*
