# Supplementary figures and images for: Regulation of submaxillary gland androgen-regulated protein 3A via estrogen receptor 2 in radioresistant head and neck squamous cell carcinoma cells
Source: J Exp Clin Cancer Res. 2017 Feb 6;36:25. doi: 10.1186/s13046-017-0496-2 (PMC5294868; doi:10.1186/s13046-017-0496-2)

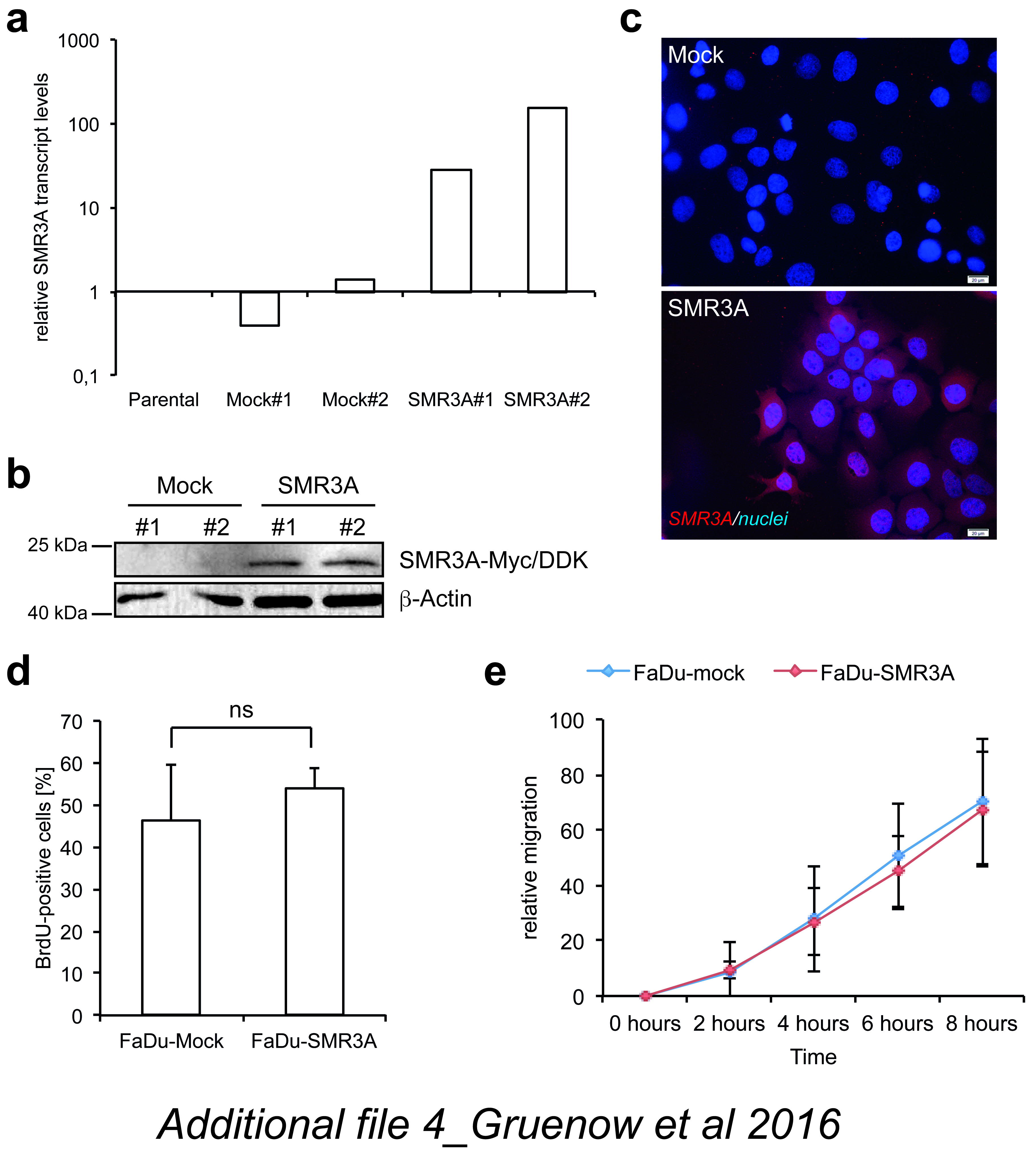

Supplement: Additional file 4: — No impact of ectopic SMR3A expression in FaDu cells on tumor-relevant processes under normal growth conditions. (a) Graph represents relative SMR3A transcript levels as determined by RQ-PCR with cDNA from parental FaDu cells (set to one), two mock controls (Mock #1 and #2) and two clones with stable SMR3A transgene expression (SMR3A#1 and #2) derived thereof. Bars represent mean values of an experiment performed in triplicates with quantification of LMNB1 transcript levels as reference gene. SMR3A-Myc/DDK transgene expression in FaDu-SMR3A clones was confirmed by Western blot analysis using an anti-Myc antibody (b) and immunofluorescence staining (red signal) with an anti-SMR3A antibody (c). Detection of β-Actin served as control for protein quantity and quality, and cell nuclei were visualized by counterstained with Hoechst H33342 (blue signal). Scale bars = 20 μm. No significant differences were detectable between FaDu-Mock and FaDu-SMR3A clones considering proliferation as determined by BrdU incorporation (d) or migration as determined by an ibidi Culture Inserts assay (e). Graphs represent mean values ± SEM of three independent experiments. (TIF 7232 kb) [file 13046_2017_496_MOESM4_ESM.tif]

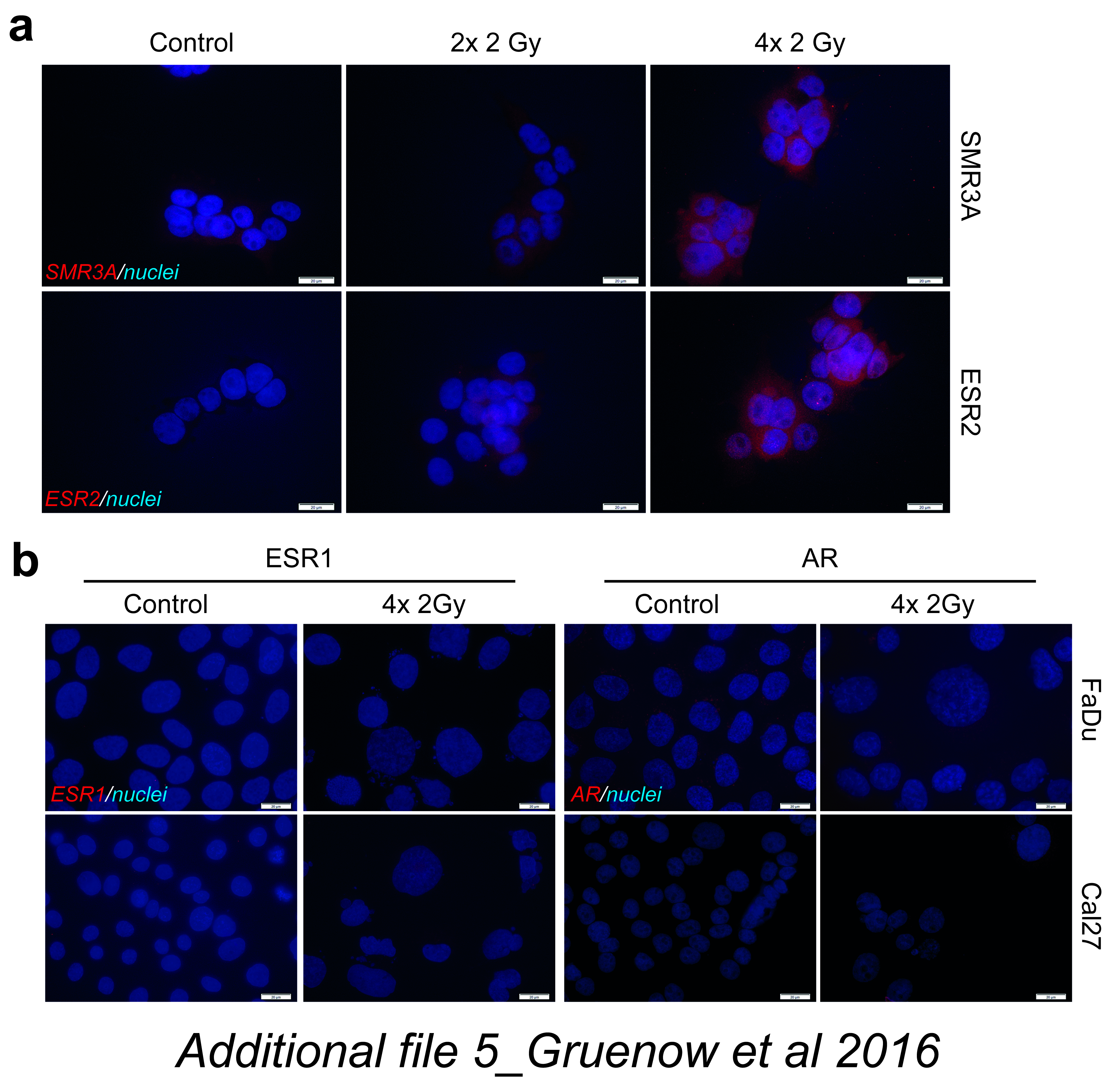

Supplement: Additional file 5: — Co-expression of SMR3A and ESR2 in FaDu cells after fractionated IR. Representative pictures of immunofluorescence staining for control and irradiated FaDu cells demonstrate prominent SMR3A and ESR2 expression a (red signal), but no positive staining for ESR1 or AR b Cell nuclei were visualized by counterstained with Hoechst H33342 (blue signal). Scale bars = 20 μm. (TIF 16501 kb) [file 13046_2017_496_MOESM5_ESM.tif]

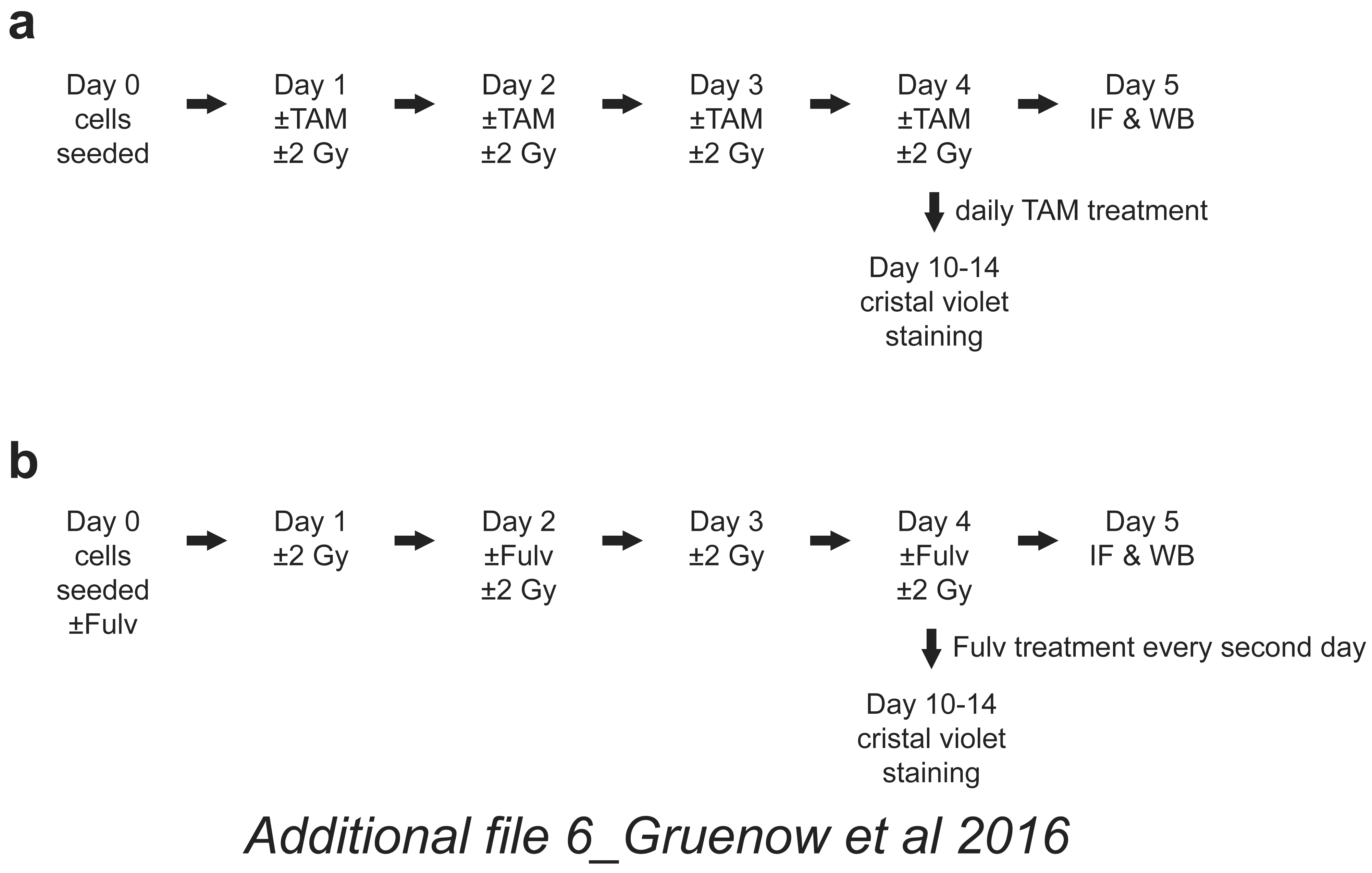

Supplement: Additional file 6: — Schematic summary of the treatment protocol for fractionated IR in combination with 4-Hydroxytamoxifen (TAM, a) or Fulvestrant (Fulv, b). (TIF 277 kb) [file 13046_2017_496_MOESM6_ESM.tif]
