# Supplementary material for: Regulation of submaxillary gland androgen-regulated protein 3A via estrogen receptor 2 in radioresistant head and neck squamous cell carcinoma cells
Source: J Exp Clin Cancer Res. 2017 Feb 6;36:25. doi: 10.1186/s13046-017-0496-2 (PMC5294868; doi:10.1186/s13046-017-0496-2)
Supplement: Additional file 7: — Correlation of ESR2 expression with histopathological and clinical characteristics. (DOCX 70 kb) [file 13046_2017_496_MOESM7_ESM.docx]

**Additional file 7. Correlation of ESR2 expression with histopathological and clinical characteristics**

|  |  | **ESR2^neg^** | | **ESR2^pos^** | |  |
| --- | --- | --- | --- | --- | --- | --- |
| **Features** | **Category** | **N** | **%** | **N** | **%** | **p value**^3^ |
| Age [years] | < 57.5 | 25 | 65.8 | 30 | 42.3 | **0.019** |
|  | ≥ 57.5 | 13 | 34.2 | 41 | 57.7 |  |
| Gender | Male | 33 | 86.8 | 50 | 71.4 | 0.055 |
|  | Female | 5 | 13.2 | 21 | 29.6 |  |
| T status | T1-T2 | 10 | 26.3 | 33 | 46.5 | **0.040** |
|  | T3-T4 | 28 | 73.7 | 38 | 53.5 |  |
| N status | N0 | 4 | 10.5 | 8 | 11.3 | 0.906 |
|  | N+ | 34 | 89.5 | 63 | 88.7 |  |
| M status | M0 | 35 | 92.1 | 66 | 95.7 | 0.445 |
|  | M+ | 3 | 7.9 | 3 | 4.3 |  |
| Pathological grading | G1-2 | 17 | 54.8 | 35 | 57.4 | 0.816 |
|  | G3 | 14 | 45.2 | 26 | 42.6 |  |
| Clinical staging | I-III | 8 | 21.1 | 21 | 29.6 | 0.337 |
|  | IV | 30 | 78.9 | 50 | 70.4 |  |
| Alcohol | no/former | 5 | 13.2 | 14 | 19.7 | 0.390 |
|  | current | 33 | 86.8 | 57 | 80.3 |  |
| Tobacco | no/former | 5 | 13.2 | 22 | 31.0 | **0.040** |
|  | current | 33 | 86.8 | 49 | 69.0 |  |
| HPV | non-related^1^ | 31 | 81.6 | 50 | 74.6 | 0.415 |
|  | related^2^ | 7 | 18.4 | 17 | 25.4 |  |
| Therapy | adjuvant RT & RCT | 22 | 57.9 | 49 | 69.0 | 0.246 |
|  | definitive RT & RCT | 16 | 42.1 | 22 | 31.0 |  |
|  | all RT | 28 | 73.7 | 44 | 62.0 | 0.218 |
|  | all RCT | 10 | 26.3 | 27 | 38.0 |  |

*RT, radiotherapy, RCT, radiochemotherapy, ^1^ viral DNA-negative or DNA-positive but transcript-negative; ^2^ viral DNA- and transcript-positive according to Holzinger et al., 2012; ^3^ Chi-square test.*
