# Supplementary material for: Regulation of submaxillary gland androgen-regulated protein 3A via estrogen receptor 2 in radioresistant head and neck squamous cell carcinoma cells
Source: J Exp Clin Cancer Res. 2017 Feb 6;36:25. doi: 10.1186/s13046-017-0496-2 (PMC5294868; doi:10.1186/s13046-017-0496-2)
Supplement: Additional file 8: — Univariate Cox regression models for progression-free and disease-specific survival. (DOCX 77 kb) [file 13046_2017_496_MOESM8_ESM.docx]

**Additional file 8. Univariate Cox regression models for progression-free and disease-specific survival**

|  | **Progression-free survival** | | | **Disease-specific survival** | | |
| --- | --- | --- | --- | --- | --- | --- |
| **Risk factor** | **HR** | **95% CI** | **p-value** | **HR** | **95% CI** | **p-value** |
| Gender  female vs male^1^ | 1.111 | 0.626-1.973 | 0.719 | 1.139 | 0.627-2.069 | 0.668 |
| Age [years]  ≥58 vs <58^1^ | 0.647 | 0.403-1.039 | 0.072 | 0.715 | 0.433-1.180 | 0.189 |
| T status  T3-4 vs T1-2^1^ | **1.831** | **1.097-3.055** | **0.021** | **2.189** | **1.238-3.871** | **0.007** |
| N status  N+ vs N0^1^ | 0.981 | 0.486-1.980 | 0.957 | 1.090 | 0.518-2.296 | 0.820 |
| Pathological grading  G3 vs G1-2^1^ | 1.074 | 0.627-1.840 | 0.795 | 0.985 | 0.551-1.762 | 0.961 |
| Clinical staging  IV vs I-III^1^ | 1.394 | 0.814-2.387 | 0.226 | 1.842 | 0.998-3.401 | 0.051 |
| Alcohol  current vs never/former^1^ | 1.073 | 0.563-2.043 | 0.831 | 1.187 | 0.603-2.337 | 0.620 |
| Tobacco  current vs never/former^1^ | **2.868** | **1.466-5.617** | **0.002** | **2.606** | **1.284-5.290** | **0.008** |
| HPV status^2^  related vs non-related^1^ | **0.264** | **0.120-0.577** | **0.001** | **0.282** | **0.121-0.655** | **0.003** |
| Subgroup  ESR2^pos^SMR3A^high^ vs ESR2^pos^SMR3A^low,1^ | **2.514** | **1.333-4.739** | **0.004** | **2.442** | **1.260-4.730** | **0.008** |
| Subgroup  All others vs ESR2^pos^SMR3A^low,1^ | **2.492** | **1.469-4.230** | **0.001** | **2.307** | **1.319-4.036** | **0.003** |

*HR = Hazard ratio, CI = confidence interval, ^1^reference group, ^2^related = viral DNA^+^RNA^+^, non-related = viral DNA^+^RNA^-^ or viral DNA^-^ according to Holzinger et al., 2012.*
