# Supplementary material for: Regulation of submaxillary gland androgen-regulated protein 3A via estrogen receptor 2 in radioresistant head and neck squamous cell carcinoma cells
Source: J Exp Clin Cancer Res. 2017 Feb 6;36:25. doi: 10.1186/s13046-017-0496-2 (PMC5294868; doi:10.1186/s13046-017-0496-2)
Supplement: Additional file 9: — Multivariable Cox regression models for progression-free and disease-specific survival. (DOCX 107 kb) [file 13046_2017_496_MOESM9_ESM.docx]

**Additional file 9. Multivariable Cox regression models for progression-free and disease-specific survival**

|  | **Progression-free survival** | | | **Disease-specific survival** | | |
| --- | --- | --- | --- | --- | --- | --- |
| **Risk factor** | **HR** | **95% CI** | **p-value** | **HR** | **95% CI** | **p-value** |
| Gender  female vs male^1^ | 1.109 | 0.506-2.427 | 0.796 | 1.042 | 0.476-2.280 | 0.919 |
| Age [years]  ≥58 vs <58^1^ | 0.618 | 0.295-1.296 | 0.209 | 0.624 | 0.280-1.394 | 0.250 |
| Clinical staging  IV vs. I-III^1^ | 1.530 | 0.745-3.142 | 0.247 | **1.966** | **0.909-4.251** | **0.086** |
| Alcohol  current vs never/former^1^ | 1.166 | 0.493-2.760 | 0.726 | 1.490 | 0.612-3.631 | 0.380 |
| Tobacco  current vs never/former^1^ | 2.016 | 0.720-5.646 | 0.182 | 1.799 | 0.639-5.011 | 0.268 |
| HPV status^2^  related vs non-related^1^ | 0.429 | 0.130-1.480 | 0.184 | 0.415 | 0.124-1.388 | 0.153 |
| Therapy  RCT vs RT^1^ | 0.700 | 0.308-1.591 | 0.395 | 0.713 | 0.295-1.725 | 0.453 |
| Subgroup  ESR2^pos^SMR3A^high^ vs ESR2^pos^SMR3A^low,1^ | **1.965** | **0.960-4.025** | **0.065** | **1.988** | **0.912-4.333** | **0.084** |

*N=66 cases were included based on complete clinical data, HR = Hazard ratio, CI = confidence interval, ^1^reference group, ^2^related = viral DNA^+^RNA^+^, non-related = viral DNA^+^RNA^-^ or viral DNA^-^ according to Holzinger et al., 2012.*

|  | **Progression-free survival** | | | **Disease-specific survival** | | |
| --- | --- | --- | --- | --- | --- | --- |
| **Risk factor** | **HR** | **95% CI** | **p-value** | **HR** | **95% CI** | **p-value** |
| Gender  female vs male^1^ | 1.004 | 0.554-1.822 | 0.999 | 0.959 | 0.519-1.770 | 0.893 |
| Age [years]  ≥58 vs <58^1^ | 0.686 | 0.421-1.118 | 0.130 | 0.719 | 0.426-1.213 | 0.216 |
| Clinical staging  IV vs. I-III^1^ | 1.544 | 0.876-2.721 | 0.133 | **2.039** | **1.081-3.843** | **0.028** |
| Alcohol  current vs never/former^1^ | 1.077 | 0.516-1.965 | 0.983 | 1.237 | 0.623-2.455 | 0.544 |
| Tobacco  current vs never/former^1^ | **1.855** | **0.922-3.734** | **0.083** | 1.783 | 0.854-3.722 | 0.124 |
| HPV status^2^  related vs non-related^1^ | **0.298** | **0.134-0.659** | **0.003** | **0.308** | **0.130-0.727** | **0.007** |
| Therapy  RCT vs RT^1^ | 0.928 | 0.543-1.584 | 0.783 | 0.943 | 0.532-1.675 | 0.842 |
| Subgroup  All others vs ESR2^pos^SMR3A^low,1^ | **1.877** | **1.075-3.277** | **0.027** | 1.561 | 0.872-2.796 | 0.134 |

*N=103 cases were included based on complete clinical data, HR = Hazard ratio, CI = confidence interval, ^1^reference group, ^2^related = viral DNA^+^RNA^+^, non-related = viral DNA^+^RNA^-^ or viral DNA^-^ according to Holzinger et al., 2012.*
